# Supplementary material for: Allosteric regulation and crystallographic fragment screening of SARS-CoV-2 NSP15 endoribonuclease
Source: Nucleic Acids Res. 2023 Apr 28;51(10):5255–70. doi: 10.1093/nar/gkad314 (PMC10250223; doi:10.1093/nar/gkad314)
Supplement: gkad314_Supplemental_Files [file gkad314_supplemental_files.zip › Supplementary datasets.zip!/217/Refine_9/01-BUSTER/Cycle-5/shell.01/R_Rfree.html]

# Rxpct factors in resolution bins at cycle 0


|  |  |  |  |  |  |  |  |  |
| --- | --- | --- | --- | --- | --- | --- | --- | --- |
|  | **<d> (Å)**  Rxpct\_Work | Rxpct\_Free | Rxpct\_All | |  |  |  |  |
|  | 16.1098 | 0.3795 | 0.4374 | 0.3815 |  |
|  | 9.3010 | 0.2275 | 0.2744 | 0.2303 |  |
|  | 7.2045 | 0.1985 | 0.2683 | 0.2024 |  |
|  | 6.0889 | 0.2002 | 0.2685 | 0.2033 |  |
|  | 5.3699 | 0.1573 | 0.2404 | 0.1607 |  |
|  | 4.8573 | 0.1440 | 0.1931 | 0.1468 |  |
|  | 4.4681 | 0.1463 | 0.2193 | 0.1500 |  |
|  | 4.1595 | 0.1449 | 0.2127 | 0.1488 |  |
|  | 3.9072 | 0.1558 | 0.2322 | 0.1596 |  |
|  | 3.6958 | 0.1598 | 0.2300 | 0.1629 |  |
|  | 3.5155 | 0.1676 | 0.1997 | 0.1692 |  |
|  | 3.3591 | 0.1734 | 0.2326 | 0.1768 |  |
|  | 3.2220 | 0.1864 | 0.2279 | 0.1887 |  |
|  | 3.1003 | 0.1921 | 0.2205 | 0.1934 |  |
|  | 2.9915 | 0.1938 | 0.2414 | 0.1956 |  |
|  | 2.8934 | 0.2063 | 0.2512 | 0.2086 |  |
|  | 2.8044 | 0.2074 | 0.2360 | 0.2086 |  |
|  | 2.7231 | 0.2090 | 0.2730 | 0.2122 |  |
|  | 2.6484 | 0.2393 | 0.2964 | 0.2430 |  |
|  | 2.5796 | 0.2565 | 0.2701 | 0.2573 |  |
|  | **Overall** | 0.1847 | 0.2394 | 0.1875 |  |

# Rxpct factors in resolution bins at cycle 1


|  |  |  |  |  |  |  |  |  |
| --- | --- | --- | --- | --- | --- | --- | --- | --- |
|  | **<d> (Å)**  Rxpct\_Work | Rxpct\_Free | Rxpct\_All | |  |  |  |  |
|  | 16.1098 | 0.3802 | 0.4376 | 0.3821 |  |
|  | 9.3010 | 0.2270 | 0.2751 | 0.2299 |  |
|  | 7.2045 | 0.1986 | 0.2691 | 0.2025 |  |
|  | 6.0889 | 0.2000 | 0.2703 | 0.2032 |  |
|  | 5.3699 | 0.1573 | 0.2402 | 0.1607 |  |
|  | 4.8573 | 0.1439 | 0.1929 | 0.1467 |  |
|  | 4.4681 | 0.1462 | 0.2194 | 0.1499 |  |
|  | 4.1595 | 0.1449 | 0.2129 | 0.1488 |  |
|  | 3.9072 | 0.1558 | 0.2324 | 0.1596 |  |
|  | 3.6958 | 0.1598 | 0.2300 | 0.1629 |  |
|  | 3.5155 | 0.1676 | 0.1999 | 0.1692 |  |
|  | 3.3591 | 0.1733 | 0.2327 | 0.1768 |  |
|  | 3.2220 | 0.1864 | 0.2279 | 0.1887 |  |
|  | 3.1003 | 0.1921 | 0.2206 | 0.1935 |  |
|  | 2.9915 | 0.1937 | 0.2413 | 0.1956 |  |
|  | 2.8934 | 0.2063 | 0.2512 | 0.2086 |  |
|  | 2.8044 | 0.2073 | 0.2359 | 0.2086 |  |
|  | 2.7231 | 0.2090 | 0.2730 | 0.2122 |  |
|  | 2.6484 | 0.2393 | 0.2965 | 0.2430 |  |
|  | 2.5796 | 0.2565 | 0.2702 | 0.2573 |  |
|  | **Overall** | 0.1847 | 0.2396 | 0.1875 |  |

# Rxpct factors in resolution bins at cycle 2


|  |  |  |  |  |  |  |  |  |
| --- | --- | --- | --- | --- | --- | --- | --- | --- |
|  | **<d> (Å)**  Rxpct\_Work | Rxpct\_Free | Rxpct\_All | |  |  |  |  |
|  | 16.1098 | 0.3801 | 0.4376 | 0.3821 |  |
|  | 9.3010 | 0.2270 | 0.2751 | 0.2298 |  |
|  | 7.2045 | 0.1986 | 0.2692 | 0.2025 |  |
|  | 6.0889 | 0.2000 | 0.2702 | 0.2032 |  |
|  | 5.3699 | 0.1572 | 0.2402 | 0.1607 |  |
|  | 4.8573 | 0.1439 | 0.1930 | 0.1467 |  |
|  | 4.4681 | 0.1462 | 0.2195 | 0.1499 |  |
|  | 4.1595 | 0.1449 | 0.2129 | 0.1488 |  |
|  | 3.9072 | 0.1558 | 0.2324 | 0.1596 |  |
|  | 3.6958 | 0.1599 | 0.2300 | 0.1630 |  |
|  | 3.5155 | 0.1676 | 0.1999 | 0.1692 |  |
|  | 3.3591 | 0.1733 | 0.2326 | 0.1768 |  |
|  | 3.2220 | 0.1864 | 0.2279 | 0.1887 |  |
|  | 3.1003 | 0.1921 | 0.2206 | 0.1935 |  |
|  | 2.9915 | 0.1938 | 0.2413 | 0.1956 |  |
|  | 2.8934 | 0.2062 | 0.2512 | 0.2086 |  |
|  | 2.8044 | 0.2074 | 0.2359 | 0.2086 |  |
|  | 2.7231 | 0.2090 | 0.2731 | 0.2123 |  |
|  | 2.6484 | 0.2393 | 0.2966 | 0.2430 |  |
|  | 2.5796 | 0.2566 | 0.2705 | 0.2574 |  |
|  | **Overall** | 0.1847 | 0.2396 | 0.1875 |  |

# Rxpct factors in resolution bins at cycle 3


|  |  |  |  |  |  |  |  |  |
| --- | --- | --- | --- | --- | --- | --- | --- | --- |
|  | **<d> (Å)**  Rxpct\_Work | Rxpct\_Free | Rxpct\_All | |  |  |  |  |
|  | 16.1098 | 0.3757 | 0.4286 | 0.3775 |  |
|  | 9.3010 | 0.2248 | 0.2743 | 0.2277 |  |
|  | 7.2045 | 0.1965 | 0.2637 | 0.2002 |  |
|  | 6.0889 | 0.1986 | 0.2618 | 0.2015 |  |
|  | 5.3699 | 0.1566 | 0.2492 | 0.1604 |  |
|  | 4.8573 | 0.1432 | 0.1971 | 0.1463 |  |
|  | 4.4681 | 0.1457 | 0.2229 | 0.1496 |  |
|  | 4.1595 | 0.1448 | 0.2145 | 0.1488 |  |
|  | 3.9072 | 0.1554 | 0.2307 | 0.1591 |  |
|  | 3.6958 | 0.1597 | 0.2309 | 0.1628 |  |
|  | 3.5155 | 0.1682 | 0.2014 | 0.1698 |  |
|  | 3.3591 | 0.1738 | 0.2288 | 0.1770 |  |
|  | 3.2220 | 0.1866 | 0.2296 | 0.1890 |  |
|  | 3.1003 | 0.1926 | 0.2181 | 0.1938 |  |
|  | 2.9915 | 0.1940 | 0.2427 | 0.1958 |  |
|  | 2.8934 | 0.2060 | 0.2510 | 0.2084 |  |
|  | 2.8044 | 0.2078 | 0.2364 | 0.2091 |  |
|  | 2.7231 | 0.2099 | 0.2750 | 0.2132 |  |
|  | 2.6484 | 0.2392 | 0.3043 | 0.2434 |  |
|  | 2.5796 | 0.2562 | 0.2751 | 0.2572 |  |
|  | **Overall** | 0.1842 | 0.2399 | 0.1870 |  |
